# Functional Fear Predicts Public Health Compliance in the COVID-19 Pandemic

**DOI:** 10.1007/s11469-020-00281-5

**Published:** 2020-04-27

**Authors:** Craig A. Harper, Liam P. Satchell, Dean Fido, Robert D. Latzman

**Affiliations:** 1 Department of Psychology, Nottingham Trent University, 50 Shakespeare Street, Nottingham, NG1 4FQ UK; 2 Department of Psychology, University of Winchester, Winchester, UK; 3 College of Business, Law & Social Sciences, University of Derby, Derby, UK; 4 Department of Psychology, Georgia State University, Atlanta, GA USA

**Keywords:** COVID-19, Pandemic response, Health anxiety, Social distancing, Hand hygiene, Public health

## Abstract

In the current context of the global pandemic of coronavirus disease-2019 (COVID-19), health professionals are working with social scientists to inform government policy on how to slow the spread of the virus. An increasing amount of social scientific research has looked at the role of public message framing, for instance, but few studies have thus far examined the role of individual differences in emotional and personality-based variables in predicting virus-mitigating behaviors. In this study, we recruited a large international community sample (*N* = 324) to complete measures of self-perceived risk of contracting COVID-19, fear of the virus, moral foundations, political orientation, and behavior change in response to the pandemic. Consistently, the only predictor of positive behavior change (e.g., social distancing, improved hand hygiene) was fear of COVID-19, with no effect of politically relevant variables. We discuss these data in relation to the potentially functional nature of fear in global health crises.

Originating in December 2019 in the Hubei province of China, the coronavirus disease-2019 (COVID-19) has spread to most countries in the world. At the time of writing (March 28, 2020), estimates of the fatality rate differ across countries and range from 0.3% in Singapore to 11% in Wuhan, China (Chen et al. 2020). However, a more recent analysis of the mortality rate in locations with early quarantine procedures (e.g., the Diamond Princess cruise ship) suggests that the true rate to be no more than 1% (Rajgor et al. 2020). While the symptoms of COVID-19 mimic the conditions caused by other coronaviruses (e.g., coughing, fever, and fatigue; Wang et al. 2020), many infected individuals can appear asymptomatic (Holshue et al. 2020), and thus can unwittingly transmit the virus to others in their vicinity. This claim was supported through a comparison of confirmed case rates between South Korea (who adopted widespread public testing for COVID-19 in February 2020) and Italy (who tested only those with symptoms), with Backhaus (2020) reporting how, in South Korea, substantially higher rates of younger people (below the age of 30) tested positive for the virus in the absence of noticeable symptoms.

As demonstrated by the differences in testing practices mentioned above, countries across the globe have taken different steps to contain and delay the spread of the virus within their borders, with differing degrees of success. China, for example, appeared to have halted the exponential increase of confirmed cases of the virus by limiting the freedom of citizens to move around their cities, provinces, and the country as a whole (Graham-Harrison and Kuo 2020). The Italian government has implemented a similar strategy, placing much of the country into lockdown and preventing groups of people from congregating in public spaces. Other countries have been slower to respond with such drastic action. The USA, for example, waited more than 2 weeks after the first confirmed case within its borders to enact localized testing procedures (Baird 2020). Elsewhere, the British government followed the instructions of its scientific advisors to delay the cancellation of mass gatherings and the closure of schools, instead favoring a policy that looked akin to pursuing herd immunity (whereby vulnerable groups are isolated, and healthier individuals are exposed to the virus to gain immunity at the population level; Fine et al. 2011).

The one common piece of advice across different countries is that those who display symptoms of COVID-19 should self-isolate from others for a period of 7–14 days, while those without symptoms (who, importantly, could still be infected) should practice “social distancing” (World Health Organization 2020). This means that everybody should be limiting non-essential social interactions, not travel unless absolutely necessary, and work from home wherever possible to slow the interpersonal transmission of the virus. However, this is being practiced inconsistently in the absence of government enforcement. In this paper, we explore some of the potential psychological triggers for this inconsistency in social distancing behavior.

In spite of us still only being in the early stage of the COVID-19 pandemic, a rapidly growing body of research into social responses to the virus is emerging. This research examines how to encourage prosocial and virus-mitigating responses (e.g., social distancing, or the non-hoarding of food and household supplies, and good hand hygiene). For example, Everett et al. (2020) reported how communicating advice using deontological moral advice (i.e., in ways that invoke a sense of civic duty) has a modest effect on increasing a propensity to engage in behaviors that enhance a delay in the transmission of the virus (e.g., hand washing, avoiding large gatherings, and sharing government advice on preventing the spread of COVID-19). This sense of duty taps into themes of interpersonal empathy, with Oosterhoff and Palmer (2020) findings that American adolescents who demonstrated higher levels of subjective self-interest were more likely to hoard supplies and less likely to refrain from non-essential social interactions. Pfattheicher et al. (2020) reported how trait empathy for vulnerable members of the population encourages the reduction of physical social interactions, while inducing empathy maintains this behavior. Further, a rapid review of the evidence into compliance with quarantine advice during pandemics recently reported that appealing to altruistic motivations to comply with distancing instructions appears to maintain motivation to maintain social distance from others over an extended period of time (Brooks et al. 2020).

While this raft of empirical work appears to be focused around persuading the community to follow social distancing advice, there may also be intrinsic or individual difference reasons for following such isolating practices. Moral foundations theory (for a review, see Haidt and Joseph 2004) asserts that individuals make social and political judgments based on their endorsement of propositions rooted in a finite set of intuitive moral domains. In the main, political liberals value making decisions on the basis of care/harm (i.e., protection of the vulnerable) and fairness/reciprocity (i.e., proportionality) principles, while political conservatives also value authority/respect, ingroup/loyalty, and purity/sanctity. There is a case to be made that these moral foundations may play a role in decisions to engage in advice about delaying the spread of COVID-19. For example, framing of vulnerability among certain demographic groups (e.g., the elderly, pregnant, and chronically ill) has the potential to trigger the care/harm foundation. Further, the advice coming from governmental or scientifically authoritative sources may trigger instincts related to the authority foundation, while the infectious nature of the virus, by definition, is likely to trigger a behavioral response associated with pathogen avoidance via the disgust foundation. For these reasons, it is unlikely that “political orientation” as a composite or self-identified variable would predict behavioral responses to COVID-19. However, specific moral foundations that are associated with positions across the ideological spectrum may differentially predict actions that mitigate the risk of the virus being spread.

According to Ahorsu et al. (2020), one unique feature of pandemic viral infections is the fear that they can instill across large swathes of the population. Fear is a negative emotion symptomized by extreme levels of emotive avoidance in relation to specific stimuli (Perin et al. 2015). It is associated with clinical phobias and social anxiety disorder (Krueger et al. 2018), and as such the potential for widespread public fear caused by pandemic viral infections could lead to significant levels of mental distress at the population level. This was implicated in a tweet published by Dr. David Murphy (president of the British Psychological Society) that highlighted fear and anxiety (operationalized as managing health anxiety and coping with worry about relatives) as targets for psychological input within the COVID-19 pandemic (Murphy 2020). In spite of the documented negative wellbeing implications of fear and anxiety, these negative emotions do also motivate a range of behaviors that reduce the engagement in risky behaviors. For example, early levels of anxiety in children are associated with lower levels of alcohol use (Kaplow et al. 2001) and cigarette and marijuana use (Colder et al. 2013). Further, pathological low levels of fear are widely associated with psychopathic personality traits (see Patrick et al. 2009; Patrick and Drislane 2015), and this has been related to various risk-taking for social and recreational purposes (Satchell et al. 2018). This is also consistent with emerging evidence that higher levels of so-called dark personality traits (e.g., psychopathy, meanness, and disinhibition) are associated with a lack of engagement with health-promoting behaviors in the COVID-19 pandemic (Blagov 2020). As such, the potential utility of a fear or anxiety response in the current context should be explored.

Specifically in relation to COVID-19, while perceiving the virus to be severe has been linked with worse mental health outcomes (Li et al. 2020), feeling personally at risk of infection predicted a greater propensity to engage in hand washing and social distancing behaviors in the early stages of the pandemic (Wise et al. 2020). Even among the aforementioned Li et al. (2020) research, subjectively judged self-control attenuated the link between perceived COVID-19 severity and poorer mental health, suggesting that combining a sensible level of fear about the illness with messages related to personal agency could encourage safety-promoting behavior in the form of hand hygiene and social distancing. This claim is supported by Zettler et al. (2020), who reported that the HEXACO personality domain of emotionality (characterized by exaggerated levels of anxiety, fear, and emotional reactivity) was associated with a greater level of acceptance of government-mandated personal restrictions. Further, Kuper-Smith et al. (2020) found that community members in the USA, the UK, and Germany consistently underestimated their likelihood of both becoming infected and transmitting COVID-19 in comparison with estimates about the “average” member of the public (see also Raude et al. 2020). They also reported negative correlations between self-perceived likelihood of infecting others and engaging in hygiene-related behaviors (e.g., hand washing and social distancing), suggesting that increasing fears about contracting the virus might lead to less risky social behaviors.

In this study, we explored multiple predictors of engaging in virus-mitigating behaviors within the context of COVID-19. Specifically, we were interested in measuring concrete behaviors in response to the COVID-19 pandemic as they occur, and predicting these using established psychological constructs. Secondary to this, we explored whether fear of COVID-19 and self-perceived likelihood of contracting the virus were associated with risk-mitigating behaviors. This is in response to an apparent mismatch between established psychopathological research into the potential utility and fear and anxiety in reducing risky behaviors, and the suggestion by Ahorsu et al. (2020) that fear might be considered a unidirectional precursor to psychopathological responses within the current context. Finally, we investigated the role of political ideology in changing behaviors in response to COVID-19. In doing so, we acknowledged the partisan nature of some social attitudes toward the virus itself (Pennycook et al. 2020) and governmental responses to the pandemic (see Pepinsky 2020) while contemplating the potential for intuitive moral foundations to overcome these identity-based political differences.

## Methods

### Participants

To determine our target sample size, we conducted an a priori power analysis using G*Power (version 3.1.9.2). Due to the lack of previous research to inform our expected size of effects, we define our smallest effect size of interest by what the psychological literature typically observes. For example, Funder and Ozer (2019) reviewed various summative analyses of the psychology and reported *r* = .20 as the typical effect size. Thus, we set a conservative type I and II error rate both to .05, and aimed to detect *r* = .20, concluding a target sample size of *N* ≥ 320.

A total of 344 individuals clicked on the study link. Of this, *N* = 324 participants (*M*_age_ = 34.32 years, *SD* = 11.71, 50% female) met all four attention checks and were retained for analysis. In our sample, 73% reported “British” or “UK” nationality and 79% reported residence in the UK. The majority of those with complete responses had an undergraduate degree (45%) or had attained less than undergraduate degree (38%). Most participants considered themselves “medium” risk for COVID-19 (51%), and many considered themselves “low” risk (33%). On a scale of very liberal (− 2) through “centrist” (0) to very conservative (2), participants were, on average, “somewhat liberal” (*M*_Politics_ = − 0.43, *SD* = 1.00, Skew = 0.31).

These participants were recruited via Prolific, a crowdsourcing platform, whereby survey responders receive small monetary compensation for taking part in research. Participants received the equivalent of £0.45 for their time. All data collection occurred between March 27 and 28, 2020.

## Materials

### Demographics and Perceived Risk of COVID-19

Participants were asked to report their age, gender, nationality, level and years of education, self-identified political orientation (rated on a 5-point scale from “1—Very Liberal” to “5—Very Conservative”), and their current country of residence. A self-report measure of perceived risk of COVID-19 was also requested, with participants being asked to self-report whether they considered themselves “Low-,” “Medium-,” or “High-Risk” (scored from 1 to 3).

### The Fear of Coronavirus-19 Scale (FCV-19S; Ahorsu et al. 2020)

The FCV-19S consists of 7 items (e.g., “It makes me uncomfortable to think about coronavirus-19”) measuring one’s fear of COVID-19 (greater scores indicate greater fear). Participants are asked to rate their agreement with each statement on a 5-point scale from “1—Strongly Disagree” to “5—Strongly Agree.”

### YouGov Behavior Change (YGBC; YouGov Blue 2020)

Shortly after the US public health campaign against COVID-19 began, the polling group YouGov asked a sample to self-report the degree to which seven behaviors have changed over the last week. We adapted this question in light of varied governmental responses to the pandemic, and asked participants to consider their behaviors in the month prior to any official “lockdown” in their country or state. However, we used the same behaviors as on the initial YGBC measure: *hand washing*, *changed travel*, *working from home*, *stockpiling food*, *stockpiling medicine*, *child and elder care*, and *social distancing*. Participants reported the perceived change on a 4-point scale from “1—It has not changed at all” to “4—It has changed dramatically.”

### PROMIS Emotional Distress Short Forms (PROMIS-SFs; Cella et al. 2007)

We used two of the PROMIS-SF measures to examine recent (past 7 days) emotional wellbeing. One measure used 8 items to quantify Diagnostic and Statistical Manual (5th edition; DSM-5; American Psychiatric Association 2013) depression symptoms (e.g., “I felt worthless”), and the other used 7 items to measure DSM anxiety symptoms (e.g., “I felt worried”). Participants rated each item on a 5-point scale from “1—Never” to “5—Always” with higher scores indicative of higher levels of negative affect, and a greater autonomic arousal and experience of threat, respectively.

### Moral Foundations Questionnaire (MFQ-20; Graham et al. 2008)

The MFQ-20 consisted of 22 statements spanning five moral foundations (care/harm, fairness/reciprocity, authority/respect, ingroup/loyalty, purity/sanctity; four items per foundation) using two different response formats. The first section asked participants to rate the relevance of a particular domain when they make a moral decision (11 items; e.g., “Whether or not someone acted unfairly”; fairness foundation). The second section asked participants to rate their endorsement of a range of moral propositions (11 items; e.g., “I am proud of my country’s history”; loyalty foundation). Two items on the MFQ-20 were fillers: “Whether or not someone was good at math” (section 1) and “It is better to do good than to do bad” (section 2). These are designed to catch careless responding, and were not included in calculating foundation scores. Each statement was rated on a 6-point scale (scored functionally from 0 to 5). In section 1, anchor labels are “0—Not at all relevant” to “5—Extremely relevant,” while in section 2 they are “0—Strongly disagree” to “5—Strongly agree.” Responses are averaged for each moral foundation, with higher scores being indicative of greater endorsement of each respective moral domain.

### World Health Organization: Quality of Life-BREF (WHOQOL-BREF; World Health Organization 2004)

The WHOQOL-BREF measures how one feels about their quality of life and health through 26 items (e.g., “How satisfied are you with your ability to perform your daily living activities?”). Participants are asked to rate their agreement with each statement on a 5-point scale from “1—Very dissatisfied” to “5—Very satisfied.” Greater scores were indicative of greater quality of life.

### Procedure

Participants initially provided their informed consent before entering their demographic information and perceived risk of contracting COVID-19. Following this, all study questionnaires were presented in a randomized order by the survey software (Qualtrics) to reduce the likelihood of order effects influencing the quality or validity of the data collected. On average, the study took 9.52 min to complete. This procedure followed British Psychological Society ethical standards, and was approved by an institutional ethical review panel prior to data collection.

### Analysis Plan

All analysis code and data (plus a redacted version of the survey file) can be found here: https://osf.io/cek3q/?view_only=198364d59a6c40c9b68226c8cd3a84df.

The mean responses to domains were retained for analysis. We report pairwise correlations between all variables (with notable correlations highlighted when they meet a conservative *α* = .001).

To analyze psychological predictors of engagement with WHO recommended behaviors, we first built a linear model (using base R) to explore the extent to which FCV-19S scores predicted engagement with the change in behavior (YGBC) scores. Then, we tested the additive effect of the two PROMIS-SF scales and WHOQOL-BREF when they were introduced into the base model. We compared the variance explained by these two models to investigate the unique variance explained by the FCV-19S.

Next, we tested for the effect of the MFQ and political orientation on behavior change (YGTC) scores. This base model was then compared with a second model including the FCV-19S and PROMIS-SF to answer whether political orientation has a greater effect on behavior change than anxiety or fear.

## Results

### Descriptive Statistics and Correlations Between Variables

Mean scores, standard deviations, and internal consistency coefficients for all measures are reported in Table 1. Pairwise correlations between the predictor measures and YGBC and FCV-19S scores can be found in Table 2. There was a notable moderate positive correlation between increased change in behavior and fear of COVID-19, suggesting that those with higher fear scores were those who were engaging with more public health behaviors.

**Table 1:** Descriptive statistics for key variables

Measure	*M* (*SD*)	Cronbach’s *α*
Fear of Coronavirus-19 Scale (FCV-19S)	2.58 (0.88)	.88
YouGov Behavior Change	2.56 (0.59)	.66
PROMIS Depression	2.42 (0.95)	.94
PROMIS Anxiety	2.81 (0.98)	.95
Moral foundations questionnaire		
Care/harm	3.76 (0.74)	.62
Fairness/reciprocity	3.79 (0.66)	.53
Authority/respect	2.49 (1.00)	.71
Ingroup/loyalty	2.32 (0.96)	.62
Purity/sanctity	2.82 (1.09)	.74
WHO: Quality of Life		
Physical	3.85 (0.67)	.78
Psychological	3.19 (0.55)	.54
Social	3.42 (0.85)	.69
Environment	3.62 (0.62)	.77

**Table 2:** Correlations between the measures in this study and behavior change and fear of coronavirus-19

Measure	Behavior change	Fear of coronavirus-19
Fear of Coronavirus-19 Scale (FCV-19S)	*r* = .31, *p* < .001*	
PROMIS Depression	*r* = .02, *p =* .692	*r* = .49, *p* < .001*
PROMIS Anxiety	*r* = .20, *p* < .001*	*r* = .69, *p* < .001*
Political orientation	*r* = .04, *p =* .456	*r* = − .02, *p* = .774
Reported risk	*r* = .24, *p* < .001*	*r* = .31, *p* < .001*
Moral foundations questionnaire		
Care/harm	*r* = .14, *p* = .010	*r* = .20, *p* < .001*
Fairness/reciprocity	*r* = .03, *p* = .637	*r* = .08, *p =* .169
Authority/respect	*r* = .15, *p* = .009	*r* = .14, *p =* .012
Ingroup/loyalty	*r* = .17, *p* = .002	*r* = .18, *p =* .001
Purity/sanctity	*r* = .13, *p* = .020	*r* = .25, *p* < .001*
WHO: Quality of Life		
Physical	*r* = − .04, *p* = .463	*r* = − .37, *p* < .001*
Psychological	*r* = .15, *p* = .007	*r* = − .08, *p* = .131
Social	*r* = .07, *p* = .202	*r* = .03, *p* = .572
Environment	*r* = .00, *p* = .979	*r* = − .31, *p* < .001*

*Meets the conservative *p* < .001

The only other notable correlates of behavior change included a small positive correlation with PROMIS Anxiety and a small-to-moderate positive relationship with self-reported risk. That is, those who show more anxiety symptoms and believed themselves to be at risk of contracting the virus changed their behavior more.

There were moderate-to-strong correlations between the FCV-19S scale and PROMIS Anxiety and Depression, suggesting this novel measure of fear is highly related to anxiety symptomatology. Increased FCV-19S scores also moderately correlated with increased self-reported risk of contracting the virus. Physical and environmental quality of life decreased with increased fear of coronavirus. The moral foundations of care/harm and purity/sanctity were weakly-to-moderately positively related to FCV-19S scores.

### Wellbeing Predicting Behavior Change

A baseline model using FCV-19S scores to predict behavior change explained a significant amount of variance (*R*^2^_Adj_ = .10, *p* < .001), with the fear score being a positive predictor (*β* = .21, *p* < .001). An additional model, using the DSM’s PROMIS measures explained slightly more variance in behavior change (*R*^2^_Adj_ = .12, *p* < .001), but this what not significantly more variance when considering the standard of our conservative alpha correction (*F*_2,320_ = 5.45, *p* = .005). The additive model maintained FCV-19S as a positive predictor (*β* = .22, *p* < .001), alongside a negative effect of PROMIS Depression (*β* = − .15, *p* = .001) and a non-significant predictor of PROMIS Anxiety (*β* = .09, *p* = .053).

### Political and Moral Variables Predicting Behavior Change

A model predicting behavior change using the moral foundations questionnaire and participants’ political orientation explained little variance and did not meet our conservative alpha, and no predictors in this model met our significance criterion. A model adding in the FCV-19S and PROMIS scales to the base model explained significantly more variance in behavior change than moral and political orientation (*F*_2,312_ = 11.04, *p* < .001). In this model (see Table 3), the only significant predictor was the positive effect of FCV-19S.

**Table 3:** The predictors for the models using politics and moral orientation and then additionally FCV-19S and PROMIS scales to predict behavior change

	Base model	Additive model
Variance explained	*R*^2^_Adj_ = .04, *p* = .008	*R*^2^_Adj_ = .12, *p* < .001*
Predictor		
Moral foundations questionnaire		
Care/harm	*β* = .17, *p* = .015	*β* = .08, *p* = .152
Fairness/reciprocity	*β* = − .11, *p* = .094	*β* = − .06, *p* = .295
Authority/respect	*β* = .03, *p* = .534	*β* = .04, *p* = .424
Ingroup/loyalty	*β* = .08, *p* = .095	*β* = .05, *p* = .308
Purity/sanctity	*β* = .02, *p* = .720	*β* = − .03, *p* = .482
Politics	*β* = − .02, *p* = .555	*β* = − .01, *p* = .820
PROMIS Depression	-	*β* = − .13, *p* = .005
PROMIS Anxiety	-	*β* = .08, *p* = .143
Fear of Coronavirus-19 Scale (FCV-19S)	-	*β* = .20, *p* < .001*

*Meets the conservative *p* < .001

## Discussion

The current study explored psychological predictors of behavior change and fear in response to the COVID-19 pandemic of 2020. We found relationships between behavior change and the new FCV-19S scale (Ahorsu et al. 2020), DSM-based anxiety and depression measures, and self-perceived risk of contracting the virus. Critically, these relationships were generally positive, in that those participants who were more concerned about COVID-19 (as measured by the FCV-19S) were those who engaged more with public health-compliant behaviors (e.g., regular hand washing, and social distancing). It is of interest that the measures of fear and anxiety symptoms were stronger predictors than moral and political orientation, all of which explained small to no variance, potentially suggesting more emotional (rather than sociopolitical) influences on compliant behavior. There was also no notable decline in quality of life in relation to behavior change. However, fear of COVID-19 was related to decreased physical and environmental wellbeing. Overall, these results suggest that “fear” and anxiety at the current time have a functional role, and are related to increased compliance for improving public wellbeing.

### Implications of Results

The data that we have presented above lead to a number of important implications, not only for the ways in which we understand behavioral responses to pandemics but also for how we conceptualize the utility of negative emotions, which may not necessarily always be reflective of psychopathology, and the political context within which such behaviors take place. We now consider these two broad implications in turn.

#### Functional “Fear”

In the development of the FCV-19S the authors rightly state that “[w]ith high levels of fear, individuals may not think clearly and rationally when reacting to COVID-19” (Ahorsu et al. 2020, p. 2). This is true for the pure emotion of fear, which represents the reactive removal of oneself from a position of immediate risk. However, many of the items in the FCV-19S scale are pertinent to *anxiety*, a preparatory reaction to ambiguous or distant stimuli. Beyond the conceptual similarity in the wording of items, our analysis revealed a strong relationship between the FCV-19S and DSM-based measures of anxiety. This is important as fear and anxiety are behaviorally and neuroendocrinologically distinct responses (McNaughton and Corr 2008), with anxiety potentially having a functional preparative role to encounter future negative stimuli consistent with the data we have presented here. Indeed, as has been described in detail previously (for a review, see Perkins and Corr 2014), negative emotions, broadly, may have evolved to serve more adaptive and protective functions and may, in certain contexts, help to keep us safe. In the current context, this appears to be the case with negative emotions being protective (i.e., encouraging of public health-promoting behaviors) during the COVID-19 pandemic. That is, the results of the current study suggest that negative emotions in response to the current pandemic predict adaptive public health-compliant behavior change (e.g., hand washing, social distancing).

These findings, situated within the aforementioned larger literature pertaining to the oftentimes adaptive nature of the experience of negative emotions, raise serious concerns with efforts to identify “mental health issues” associated with strong emotional responses, which, for the majority of individuals, are both normative and protective. That is, for most individuals, the anxious responses being assessed by the FCV-19 may represent a normal and adaptive response to a real and present danger that one cannot fight or flee from, within the environment (i.e., the COVID-19 pandemic). Those working in the mental health field should be sensitive to the context in which behaviors emerge, especially when these behaviors exist in a culture of preparedness for coping with the new cultural and governmental demands that may be critical for personal and family survival.

Notwithstanding the above, it is important for mental health professionals to be attentive to the needs of individuals for whom highly emotional responses to the current pandemic, coupled with pre-existing risk factors (e.g., those with a history of mental illness), may result in pathological levels of negative emotions and related behaviors. Indeed, increased and prolonged exposure to a community crisis, like the pandemic we are currently experiencing, has been found to result in increased maladaptive levels of anxiety leading to unnecessary behaviors associated with increased levels of impairments within individuals as well as overburdening of community resources (Garfin et al. 2020). Overall, we believe that mental health professionals have an important role to play in supporting the wellbeing of the public in this current time. However, it is not clear how this work is supported by the classification of rational concerns about a pandemic and the labelling of functional “fear” as a psychological issue.

#### Politically Driven Responses to the Pandemic

In spite of recent reviews of the political psychological literature advocating for the presence of behavioral asymmetries between ideological groups (see, e.g., Jost 2017), we found no such evidence in our data. This is perhaps surprising given recent coverage of the COVID-19 pandemic, and reports about how attitudinal and behavioral responses to the virus were inherently partisan in nature (Pepinsky 2020).

In this previously reported work, US-based Democrats were more likely than Republicans to engage in health-promoting behaviors (e.g., regular hand washing, social distancing, and self-quarantining), and to express attitudes that were critical of governmental responses to the pandemic (e.g., that inadequate testing was available, or that more money should be made available to tackle the virus). An obvious difference in our data is the political context—slightly less than 80% of our sample were British, rather than American. However, it could be that this work reflects the stage of the pandemic in which the partisan data were collected. At the time of writing, the USA is in an earlier phase of the COVID-19 pandemic. In a manner consistent with a social intuitionist account of political and moral decision-making (Haidt 2001), it is therefore plausible that initial (and perhaps intuitive) judgments of the pandemic in the US context were guided by President Trump’s initial rejection of COVID-19 as a serious and unique public health concern (Trump 2020). These initial intuitions then led to asymmetrical responses between partisans in Pepinsky’s (2020) work: ambivalence and inaction among Republicans, and concern and derision of the response among Democrats. However, in the UK (where the majority of our participants were based), the pandemic is at a slightly more advanced stage, meaning that the seriousness of the situation is clearer. We argue that the lack of any significant political orientation or moral foundations effects on behavior change is a positive point from a social perspective, and suggests that, in times of (inter)national crisis, people can forgo their ideological commitments and behave consistently with governmental advice in pursuit of a common public health good.

### Limitations and Future Directions

The results of this study are not without limitation. First, the data presented here are entirely self-reported and so may be subject to response biases. Specifically, virus-mitigating behavior changes such as increased hand washing, working from home, and social distancing are all targets of governmental strategy to “flatten the curve” and reduce the spread of the virus (e.g., Public Health England 2020). As such, it may be expected that this constellation of behavioral changes will be reported to a greater degree as a function of a reluctance to deviate from this normative social shift. The collection of “other-reported” data on the participants would help to mitigate this limitation. Second, while our regression models have utility for informing how public health behavioral compliance can be predicted from an individual differences perspective, it lacks the identification and understanding of any potential barriers to bringing about said change. It is here where experimental studies emerging about the response to COVID-19 (e.g., Everett et al. 2020) can be supplemented by correlational data such as ours in order to bring about a more comprehensive view of public health compliance and the effectiveness of government messaging. Finally, we are mindful that data has been collected at a single time point within an unprecedented period of time. Therefore, not only do results of this investigating warrant replication, but also future investigations should consider any potential variation in governmental guidance, policy, or social perception pertaining to COVID-19 in their discussion and interpretation of results.

### Conclusions

This paper has presented cross-sectional data demonstrating, in real time, predictors of behavior change in response to a global viral pandemic. We have shown how “fear” may be a normal and, crucially, functional response within this context. That is, scores on the recently developed FCV-19S (Ahorsu et al. 2020), which specifically measures “fear” toward the new virus, consistently predicted engaging in culturally and governmentally recommended public health behaviors (e.g., improved hand hygiene and social distancing). In light of this, we argue that researchers and mental health professionals would be mindful to consider the context within which negative emotional states are experienced before considering whether such emotional states are necessarily pathological.

Further, and in spite of the increasingly polarized nature of our political landscape, we found that politically relevant outcomes (i.e., self-identified orientation, and the endorsement of moral foundations; Graham et al. 2009) were unrelated to behavior change. These non-significant effects highlight how universal polarization in social responses between political groups is not inevitable, and that there are some issues that unite us, rather than divide. Just as other (inter)national crises have previously brought people together (e.g., Willer 2004), it seems that the COVID-19 pandemic has the potential to do the same, and bring people of opposing political positions together in a sense of common humanity.
